# Regulation and function of H3K36 di-methylation by the trithorax-group protein complex AMC

**DOI:** 10.1242/dev.163808

**Published:** 2018-04-05

**Authors:** Sigrun Schmähling, Arno Meiler, Yoonjung Lee, Arif Mohammed, Katja Finkl, Katharina Tauscher, Lars Israel, Marc Wirth, Julia Philippou-Massier, Helmut Blum, Bianca Habermann, Axel Imhof, Ji-Joon Song, Jürg Müller

**Affiliations:** 1 Max-Planck Institute of Biochemistry, Laboratory of Chromatin Biology, Am Klopferspitz 18, 82152 Martinsried, Germany; 2 Max-Planck Institute of Biochemistry, Computational Biology, Am Klopferspitz 18 82152 Martinsried, Germany; 3 Korea Advanced Institute of Science and Technology (KAIST), Department of Biological Sciences, Daejeon 34141, Korea; 4 Zentrallabor für Proteinanalytik, BioMedical Center, Ludwig-Maximilians-University Munich, Großhadernerstr. 9, 82152 Martinsried, Germany; 5 Laboratory for Functional Genome Analysis (LAFUGA), Gene Center, Ludwig-Maximilians-Universität München, Feodor-Lynen-Strasse 25, 81377 Munich, Germany

**Keywords:** Trithorax group, Ash1, MRG15, Histone H3K36 methylation, *Drosophila*

## Abstract

The *Drosophila* Ash1 protein is a trithorax-group (trxG) regulator that antagonizes Polycomb repression at HOX genes. Ash1 di-methylates lysine 36 in histone H3 (H3K36me2) but how this activity is controlled and at which genes it functions is not well understood. We show that Ash1 protein purified from *Drosophila* exists in a complex with MRG15 and Caf1 that we named AMC. In *Drosophila* and human AMC, MRG15 binds a conserved FxLP motif near the Ash1 SET domain and stimulates H3K36 di-methylation on nucleosomes. *Drosophila MRG15*-null and *ash1* catalytic mutants show remarkably specific trxG phenotypes: stochastic loss of HOX gene expression and homeotic transformations in adults. In mutants lacking AMC, H3K36me2 bulk levels appear undiminished but H3K36me2 is reduced in the chromatin of HOX and other AMC-regulated genes. AMC therefore appears to act on top of the H3K36me2/me3 landscape generated by the major H3K36 methyltransferases NSD and Set2. Our analyses suggest that H3K36 di-methylation at HOX genes is the crucial physiological function of AMC and the mechanism by which the complex antagonizes Polycomb repression at these genes.

## Abstract (precis)

**Highlighted Article:** The trithorax group protein Ash1 and its regulator MRG15 form a multiprotein complex that maintains expression of HOX and other target genes by methylating histone H3 in their chromatin.

## INTRODUCTION

In organisms ranging from yeast to humans, the chromatin spanning the transcribed region of active genes is modified by di- and tri-methylation of lysine 36 in histone H3 (H3K36me2/3). Although nucleosomes in the 5′ regions of transcribed genes are predominantly di-methylated at H3K36, nucleosomes in the 3′ region of genes mainly carry the H3K36me3 modification (Bell et al., 2007; Pokholok et al., 2005). Among the different roles ascribed to H3K36me2/3 (Venkatesh and Workman, 2013), there is accumulating evidence for two principal mechanisms by which this modification impacts on gene transcription. First, studies in yeast revealed that the H3K36me2/3 mark is recognized by the chromo barrel domain of the Eaf3 subunit (Sun et al., 2008; Xu et al., 2008) of the Rpd3S complex that, by deacetylating nucleosomes in the transcribed region, suppresses initiation of transcription at intragenic sites (Venkatesh and Workman, 2013). H3K36me2/3 is thus thought to participate in the quality control of transcription by preventing production of unwanted transcripts. Second, in metazoans, the H3K36me2/3 modification allosterically inhibits the histone methyltransferase (HMTase) activity of Polycomb Repressive Complex 2 (PRC2) and thereby prevents PRC2 from depositing H3K27me3 on H3K36me2/3-modified nucleosomes (Schmitges et al., 2011; Yuan et al., 2011). H3K36me2/3 was therefore proposed to protect transcriptionally active genes from becoming tri-methylated at H3K27 and thereby getting repressed by the Polycomb system. This antagonism between H3K36me2/3 and PRC2 is thought to be particularly crucial at developmentally regulated genes that, although active in some cells, are at the same time repressed by Polycomb in other cells of the body (Gaydos et al., 2012; Klymenko and Müller, 2004).

In yeast, all H3K36 di- and tri-methylation is generated by a single histone methyltransferase, Set2, that associates with the phosphorylated form of elongating RNA polymerase II (Krogan et al., 2003; Venkatesh and Workman, 2013; Xiao et al., 2003). In metazoans, SET2 is responsible for generating the bulk of H3K36me3, whereas NSD generates the bulk of H3K36me2 (Bell et al., 2007; Gaydos et al., 2012; Larschan et al., 2007).

Higher metazoans contain an additional SET-domain HMTase that di-methylates H3K36, called Ash1 (An et al., 2011; Dorighi and Tamkun, 2013; Tanaka et al., 2007; Yuan et al., 2011). The *absent, small, or homeotic discs 1* (*ash1*) gene in *Drosophila* was first identified because of the phenotype of *ash1* mutants, which developed into pharate adults and showed homeotic transformations in several body segments (Shearn et al., 1987). The similarity of the homeotic phenotypes of *ash1* and *trithorax* mutants led to the classification of *ash1* as a trithorax-group (trxG) regulator (Shearn, 1989). As expected from the phenotype, *ash1* mutants show loss of expression of multiple HOX genes within their normal expression domains (LaJeunesse and Shearn, 1995). However, in *ash1* mutants that also lack PRC2, HOX gene expression is restored to normal levels and, in addition, these double mutants also show widespread misexpression of HOX genes, similar to PRC2 single mutants (Klymenko and Müller, 2004). This suggested that, at least at HOX genes, Ash1 is not required for transcriptional activation per se but is needed to antagonize instalment of Polycomb repression. It is important to note that in the wild type, PRC2 and other Polycomb group (PcG) protein complexes are bound at target genes, not only in the cells in which these genes are repressed but also in the cells in which they are expressed (Bowman et al., 2014; Kang et al., 2017; Kwong et al., 2008; Langlais et al., 2012; Papp and Müller, 2006). Nevertheless, at active genes, PRC2 fails to tri-methylate H3K27 in their chromatin. In *ash1* mutants, however, PRC2 deposits H3K27me3 ectopically across the entire promoter and coding region (Papp and Müller, 2006). Such ectopic methylation by PRC2 in *ash1* mutants is also detected on polytene chromosomes, where several genomic sites show an increase in H3K27me3 immunofluorescence signal (Dorighi and Tamkun, 2013; Srinivasan et al., 2008). Moreover, genome-wide ectopic H3K27 tri-methylation is also observed in *C. elegans* mutants lacking the NSD orthologue Mes-4 (Gaydos et al., 2012). Together with the above-mentioned finding that H3K36me2/3 inhibits H3K27 methylation by PRC2 on nucleosomes *in vitro* (Schmitges et al., 2011; Yuan et al., 2011), these observations collectively suggested that Ash1 keeps HOX and possibly also other target genes active by di-methylating H3K36 in the transcribed region of their chromatin and thereby preventing H3K27me3 deposition and instalment of Polycomb repression. However, several aspects that are central to this model have remained unresolved. First, the Ash1 protein alone shows only weak HMTase activity because its SET domain is auto-inhibited (An et al., 2011). This raises the question of how Ash1 catalytic activity becomes stimulated. Second, at least in *Drosophila* tissue culture cells, the bulk of H3K36me2 is generated by NSD (Bell et al., 2007) and it is not known where and to what extent Ash1 contributes to H3K36 di-methylation, in particular at HOX target genes. Third, it is not known whether Ash1 also regulates genes other than HOX genes during *Drosophila* development.

Here, we have biochemically purified Ash1 protein complexes from *Drosophila* and characterized their activity *in vitro* and in the developing organism. Our work reveals that Ash1 HMTase activity is activated by MRG15, a subunit of the identified Ash1 complex, and we show that this complex, rather than the Ash1 protein alone, is the active form of this H3K36 methyltransferase, both *in vitro* and *in vivo*. We show that Ash1 is not needed for global H3K36 di-methylation but is essential to generate normal H3K36me2 levels at HOX and other genes that we found to be de-regulated in *ash1* mutants. The specific homeotic phenotypes of mutants that lack Ash1 or Ash1 HMTase activity establish that H3K36 di-methylation at HOX genes is a key physiological function of the Ash1 protein complex for *Drosophila* morphogenesis.

## RESULTS

### Biochemical purification and reconstitution identify MRG15 and Caf1 as Ash1 complex subunits

**Fig. 1.**
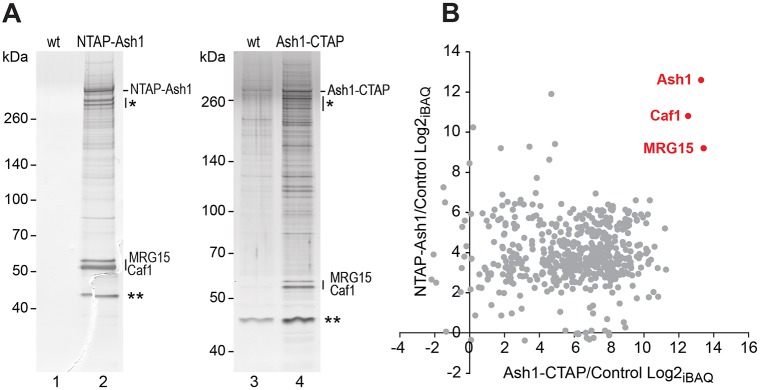
**Ash1 purified from *Drosophila* exists in a complex with MRG15 and Caf1.** (A) Proteins isolated by tandem affinity purification (TAP) from wild-type (wt) and *α-tubulin1-NTAP-Ash1* embryos (left), and from wild-type and *α-tubulin1-Ash1-CTAP* embryos (right) separated on a 4-12% polyacrylamide gel and visualized by silver staining. The bands marked by an asterisk are TAP-Ash1 degradation products; the bands marked with two asterisks were considered as non-specific because they were also detected in several mock TAPs from the wild-type control (e.g. lane 3). (B) Scatterplot representation of log2 transformed iBAQ_Ash1_/iBAQ_mock_ ratios (Intensity Based Absolute Quantification) from the mass spectrometric data of the same NTAP-Ash1 and Ash1-CTAP purifications shown in A (see Tables S1 and S2).

To identify proteins that form stable assemblies with the Ash1 protein in *Drosophila*, we used a tandem affinity purification (TAP) strategy (Rigaut et al., 1999) and purified N- or C-terminally tagged Ash1 (NTAP-Ash1 or Ash1-CTAP, respectively) from embryonic nuclear extracts (Fig. 1A). The transgenes expressing NTAP-Ash1 or Ash1-CTAP in these assays rescued animals homozygous for the *ash1^22^* null mutation (Tripoulas et al., 1996) into morphologically normal and fertile adults, permitting the purification of these fusion proteins from animals lacking untagged endogenous Ash1. Mass spectrometric analyses of the purified material identified MRG15 and Caf1-55 (for simplicity referred to as Caf1) as the two major proteins that co-purified with both NTAP-Ash1 and Ash1-CTAP (Fig. 1B). In both purifications we failed to detect Fsh1, a protein that was previously reported to co-purify with Ash1 from *Drosophila* tissue culture cells (Kockmann et al., 2013).

**Fig. 2.**
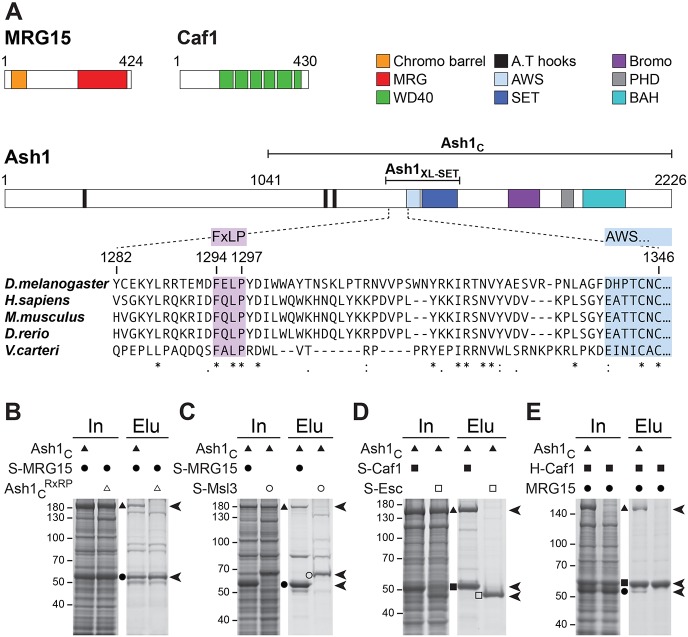
**Reconstitution of recombinant AMC and identification of the conserved Ash1 FxLP motif as the MRG15 interaction site.** (A) Domain architecture of MRG15, Caf1 and Ash1 and alignment of Ash1 protein sequences harbouring the FxLP motif. (B-E) Total extracts (In) from Hi-5 cells co-expressing the indicated AMC subunits and material isolated from these cells (Elu) by Strep- (B-D) or His- (E) affinity purification, separated on 8% (B,C,E) or 10% (D) SDS polyacrylamide gels and visualized by Coomassie Blue staining. (E) Use of the His-tag on Caf1 and a different running buffer accounts for the slower migration behaviour of Caf1 relative to MRG15 in this experiment. See text for a description of the results.

We tested whether interactions of MRG15 and Caf1 with Ash1 could be reconstituted with recombinant proteins. MRG15, like its yeast orthologue Eaf3, contains a chromo barrel domain that binds H3K36me2/3 (Zhang et al., 2006) and an MRG domain (Fig. 2A). Structural studies on MRG15 had revealed that the protein uses its MRG domain to bind extended regions of its interaction partners via high-affinity interactions that are centred around a conserved FxLP motif in those partner proteins (Xie et al., 2012, 2015). Inspection of the *Drosophila* and vertebrate Ash1 protein sequences identified in each case a single FxLP motif in a conserved location, about 40 amino acid residues N-terminal to the AWS domain that precedes the catalytic SET domain (Fig. 2A). Using baculovirus expression vectors, we co-expressed *Drosophila* Ash1_1041-2226_ (Ash1_C_) with Strep-tagged full-length MRG15 (S-MRG15) in Hi-5 insect cells and then performed Strep-affinity purification. This resulted in the isolation of an Ash1_C_:MRG15 complex (Fig. 2B). In contrast, when we co-expressed S-MRG15 with Ash1C containing a mutation of the FxLP motif to RxRP, this mutant Ash1_C_^RxRP^ protein failed to co-purify with S-MRG15 (Fig. 2B). This suggests that the FxLP motif in Ash1_C_ is crucial for interaction with MRG15. As control for the specificity of this interaction, we also tested for interaction of Ash1C with Strep-tagged Msl3 (S-Msl3). Msl3 is another MRG domain protein that uses a similar mode of interaction like MRG15 to bind a conserved FxLP motif in its binding partner MSL1 (Xie et al., 2015). However, strep affinity-purification from cells co-expressing Ash1_C_ with S-Msl3 resulted in the isolation of S-Msl3 alone (Fig. 2C). We conclude that Ash1_C_, via the conserved FxLP motif, directly and specifically interacts with MRG15.

We next investigated whether Caf1 directly interacts with Ash1_C_. Caf1 is a WD40 β-propeller protein that is a core subunit of several chromatin-modifying complexes, including PRC2, and is required for cell viability (Anderson et al., 2011). Co-expression of Ash1_C_ with S-Caf1 resulted in the isolation of an Ash1_C_:Caf1 complex. In contrast, Ash1_C_ did not co-purify with S-Esc, another WD40 β-propeller subunit of PRC2, used as control (Fig. 2D). Ash1_C_ therefore directly and specifically interacts with Caf1.

Finally, we tested whether Caf1 and MRG15 also directly bind each other. His-affinity purification from cells co-expressing His-tagged Caf1 (H-Caf1) and MRG15 resulted in the isolation of H-Caf1 alone (Fig. 2E). When Ash1_C_ was also co-expressed, a trimeric complex containing substoichiometric amounts of MRG15 could be isolated (Fig. 2E). This suggests that Caf1 and MRG15 do not interact directly and that Ash1_C_ forms a scaffold to which both proteins bind independently of each other. In conclusion, these biochemical purification and reconstitution assays identify Ash1, MRG15 and Caf1-55 as subunits of a novel protein complex that we named AMC.

### MRG15 stimulates H3K36 di-methylation in *Drosophila* and human AMC

**Fig. 3.**
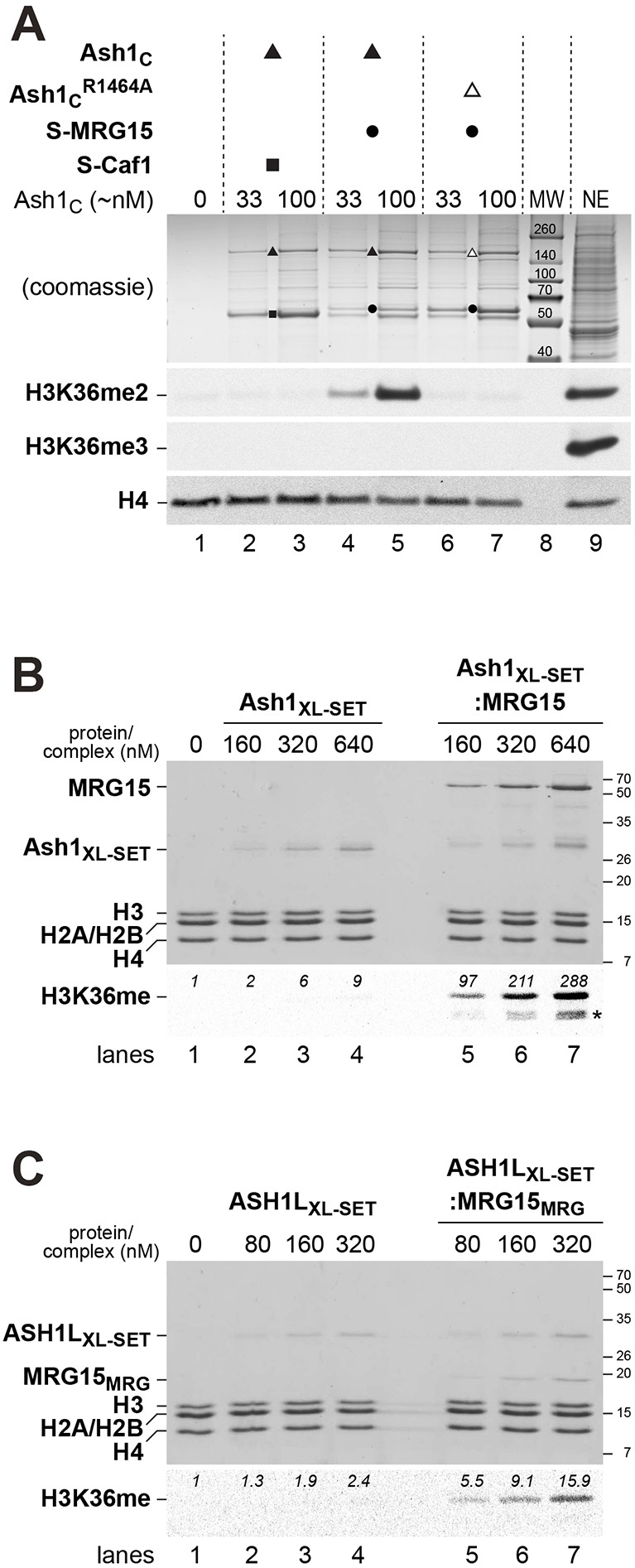
**H3K36 di-methylation by *Drosophila* Ash1 and human ASH1L is stimulated by MRG15.** (A) HMTase reactions with recombinant *Drosophila* Ash1_C_:Caf1 (lanes 2, 3), Ash1_C_:MRG15 (lanes 4, 5) or Ash1_C_^R1464A^:MRG15 (lanes 6, 7) complexes and reconstituted recombinant mononucleosomes (400 nM in lanes 1-7) were separated on a 10% SDS-polyacrylamide gel; the upper part of the gel was stained with Coomassie Blue to visualize the enzyme complexes, the bottom part was analysed by western blotting with antibodies against H3K36me2 and, as a control, H4. The same reaction was also analysed with antibody against H3K36me3. Enzyme concentrations in the reactions were normalized by estimating Ash1_C_ concentration relative to a Coomassie Blue-stained protein standard. *Drosophila* embryo nuclear extract (NE) in lane 9 served as control for western blot analysis. Lane 8: molecular weight marker (MW). (B) HMTase reactions with recombinant *Drosophila* Ash1_XL-SET_ (lanes 2-4) or Ash1_XL-SET_:MRG15 complex (lanes 5-7) on reconstituted recombinant *Xenopus* oligonucleosomes (320 nM in lanes 1-7). One part of the reaction was analysed on a 15% SDS-polyacrylamide gel to visualize proteins by Coomassie Blue staining (top), the other part of the reaction was separated on a 15% SDS-polyacrylamide gel, transferred to membrane and analysed by fluorography. PhotoShop software was used to quantify radioactive signal in the H3 band; this signal represents the sum of H3K36me1 and H3K36me2. Asterisk indicates H3 degradation products. (C) HMTase reactions with recombinant human ASH1L_XL-SET_ (lanes 2-4) or ASH1L_XL-SET_:MRG15_MRG_ complex (lanes 5-7) on reconstituted recombinant *Xenopus* oligonucleosomes (320 nM in lanes 1-7). Reactions were analysed as in B.

Previous studies reported that C-terminal fragments of *Drosophila* or mammalian Ash1 protein containing the SET domain (Fig. 2A) have HMTase activity for dimethylation of H3K36 in nucleosomes (An et al., 2011; Eram et al., 2015; Miyazaki et al., 2013; Tanaka et al., 2007; Yuan et al., 2011). Structural and enzymatic studies on the isolated SET domain of human ASH1L found that this domain has only weak HMTase activity because it is auto-inhibited by a loop from the post-SET domain that blocks access to the substrate-binding pocket (An et al., 2011). In a first set of experiments, we performed HMTase assays with the Ash1_C_:MRG15 or Ash1_C_:Caf1 complexes described above (Fig. 2). As substrate, recombinant mononucleosomes were used and the reactions were monitored by western blot analysis with antibodies against H3K36me2 or H3K36me3. Under our experimental conditions, we were unable to detect HMTase activity in reactions with the Ash1_C_:Caf1 complex (Fig. 3A, lanes 2-3) but, strikingly, the Ash1_C_:MRG15 complex showed robust activity for generating H3K36me2 (Fig. 3A, lanes 4-5). Mutation of the Ash1 SET domain at Arg1464, a residue that stabilizes the orientation of the SAM-binding loop, abolished HMTase activity; the Ash1_C_^R1464A^:MRG15 complex failed to generate detectable levels of H3K36me2 (Fig. 3A, lanes 6-7). This control confirms that H3K36 di-methylation generated by the Ash1_C_:MRG15 complex is catalysed by the Ash1 SET domain. We also note that the Ash1_C_:MRG15 complex catalyses only H3K36 di- and not tri-methylation on nucleosomes (Fig. 3A). Taken together, these analyses show that association of MRG15 with Ash1 greatly enhances its catalytic activity for H3K36 di-methylation and it therefore appears that the AMC complex and not Ash1 alone is the active form of this HMTase.

We next wanted to quantify the stimulatory effect of MRG15 on Ash1 HMTase activity. To achieve this, we expressed and purified a short soluble fragment of the *Drosophila* Ash1 protein comprising the SET domain and the preceding amino acid sequences that include the FxLP motif (Ash1_1275-1522_, called Ash1_XL-SET_; Fig. 2A and Fig. 3B, lanes 2-4). Like Ash1_C_, this Ash1_XL-SET_ fragment also interacted with full-length MRG15 and could be purified as a stable Ash1_XL-SET_:MRG15 complex (Fig. 3B, lanes 5-7). We performed HMTase assays on recombinant oligonucleosome arrays using S-[methyl-^3^H] adenosylmethionine and quantified methyl-^3^H incorporation into histone H3 using fluorography (Fig. 3B). The Ash1_XL-SET_:MRG15 complex showed about 30-fold higher HMTase activity compared with Ash1_XL-SET_ alone under our assay conditions (Fig. 3B, compare lanes 5-7 with lanes 2-4).

To extend these analyses, we also investigated the interaction between human ASH1L and MRG15. Specifically, we reconstituted and purified a recombinant minimal ASH1L_XL-SET_:MRG15_MRG_ complex containing an ASH1L_XL-SET_ protein fragment (ASH1L_2035-2288_) in complex with the MRG domain (MRG15_151-362_, called MRG15_MRG_) of human MRG15 (Fig. 3C, lanes 5-7). The human ASH1L_XL-SET_:MRG15_MRG_ complex showed about a sevenfold higher HMTase activity than the ASH1L_XL-SET_ protein alone (Fig. 3C, compare lanes 5-7 with lanes 2-4). We conclude that the interaction between ASH1L and MRG15, and the stimulatory effect of MRG15 on ASH1L HMTase activity is conserved in humans. Moreover, these results also suggest that interaction of the MRG domain of MRG15 with an ASH1L fragment comprising only the SET domain and the preceding amino acid stretch with the FxLP motif (i.e. ASH1L_XL-SET_) is sufficient to activate ASH1L HMTase activity by almost an order of magnitude.

### AMC HMTase activity is required for viability and is crucial for HOX gene regulation in *Drosophila*

In the next set of experiments, we investigated the physiological role of AMC and its H3K36 di-methyltransferase activity in *Drosophila*. To this end, we generated animals that completely lacked Ash1 or MRG15 protein, or mutants expressing full-length but catalytically inactive Ash1 protein.

**Fig. 4.**
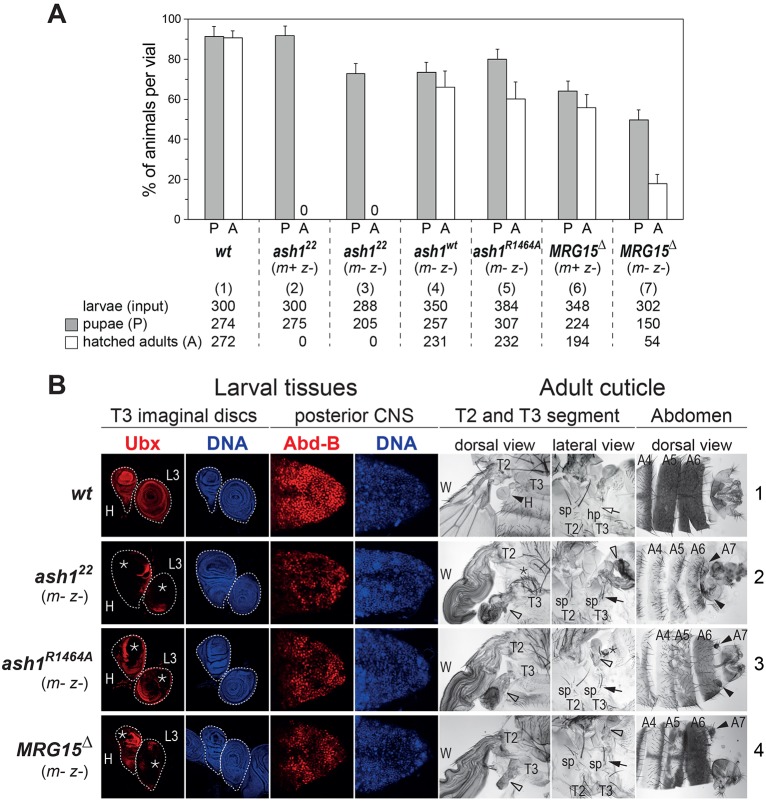
**Requirement of AMC HMTase activity for viability and HOX gene regulation.** (A) Viability of *Drosophila* with different *ash1* and *MRG15* mutant genotypes. For each genotype [(1)-(7)], indicated numbers of 1st/2nd instar larvae (input) were isolated, distributed into at least three different food vials and reared. In each vial, the percentage of animals that formed pupae (grey bar) and eclosed from the pupal case (white bar) was determined. Histograms represent the mean±s.d. of these percentages in individual vials of a given genotype. *ash1^22^* ^*m+ z−*^ and *ash1^22^* ^*m− z−*^ animals do not eclose from the pupal case (asterisk). The genotype of *MRG15*^Δ*m+ z−*^ and *MRG15*^Δ*m− z−*^ animals shown here and in B is *MRG15*^Δ^*/Df(3R)BSC741* (see text). (B) Lack of AMC function causes a specific HOX loss-of-function syndrome. Left: HOX gene expression analysis. Larval haltere (H) and third leg (L3) imaginal disc tissues stained using Ubx antibody (red) and co-stained with Hoechst to visualize nuclei (DNA, blue); CNS tissues stained using Abd-B antibody (red) and Hoechst (DNA, blue). In the wild type (wt), Ubx is expressed in all cells of the H and L3 disc. *ash1^22 m– z–^*, *ash1^R1464A m– z–^* and *MRG15*^Δ*m– z–*^ mutants show patchy loss of Ubx expression (asterisks) in irregular patterns in both discs; this phenotype is observed in all animals in all three genotypes (*n*>40) but comparison of discs from these larvae shows that the tissue area showing loss of expression is most extensive in *ash1^22 m– z–^* mutants, somewhat less extensive in *ash1^R1464A m– z–^* mutants and even less extensive in *MRG15*^Δ*m– z–*^ mutants. In wild-type animals, Abd-B is expressed in all cells of the posterior CNS, whereas *ash1^22 m– z–^*, *ash1^R1464A m– z–^* and *MRG15*^Δ*m– z–*^ mutants all show patchy loss or reduction of Abd-B expression. Right: cuticle phenotype analysis. Preparations of cuticles from adults (row 1) or pharate adults (row 2-4) with dorsal and lateral views of T2 and T3 segment structures and dorsal views of the posterior abdomen, including the A4, A5 and A6 segments. Dorsal view T2 and T3: in wild-type animals, haltere disc tissues form the haltere (H, black arrowhead) in T3. *ash1^22 m– z–^*, *ash1^R1464A m– z–^* and *MRG15*^Δ*m– z–*^ mutants show haltere-to-wing transformations (empty arrowheads; wing in T2 marked as W in all cases), owing to loss of Ubx expression. The extent of this T3 to T2 transformation matches the extent of Ubx expression loss in the three mutant genotypes (*ash1^22 m– z–^*>*ash1^R1464A m– z–^*>*MRG15*^Δ*m– z–*^). Lateral view T2 and T3: T3 to T2 transformation in *ash1^22 m– z–^*, *ash1^R1464A m– z–^* and *MRG15*^Δ*m– z–*^ mutants due to loss of Ubx expression in the 3L disc is manifested by transformation of the hypopleurite (hp) in T3 (empty arrow in wild-type animals) into sternopleurite (sp) tissue with sp bristles (black arrows), which are normally only found in T2. The haltere-to-wing (empty arrowhead) and meta- to mesonotum transformations (asterisk) are variable in animals of the same genotype (compare lateral and dorsal views). Dorsal view of abdomen: wild-type males show characteristic pigmentation in A5 and A6 that is almost completely lost in *ash1^22 m– z–^* mutants or lost in a patchy pattern in *ash1^R1464A m– z–^* and *MRG15*^Δ*m– z–*^ mutants. A7 segment structures appearing in the three mutant genotypes are indicated.

In the first experiment, we analysed the *ash1*-null mutant phenotype. Earlier studies had identified and characterized *ash1^22^* as a protein null mutation (Tripoulas et al., 1996). Consistent with these earlier reports, we were unable to detect Ash1 protein in larval extracts from *ash1^22^* homozygotes (Fig. S1). It is important to note that previous studies investigating the requirement of Ash1 during *Drosophila* development had analysed *ash1* mutant animals that were derived from heterozygous mothers and therefore contained maternally deposited wild-type Ash1 protein during the early stages of development (Shearn, 1989; Shearn et al., 1987; Tripoulas et al., 1996). The phenotype of *ash1*-null mutant animals that lack both maternally deposited and zygotically expressed Ash1 protein, in the following referred to as *ash1 ^m– z–^* mutants, has not been described. We generated *ash1^22 m– z–^* mutant animals from females with *ash1^22^* mutant germ cells. Interestingly, these *ash1^22 m– z–^* mutant animals developed up to the pupal stage and died as pharate adults, like *ash1^22 m+ z–^* mutants (Fig. 4A). *ash1^22 m– z–^* pharate adults show a spectrum of anteriorly directed homeotic transformations that are similar to but slightly more severe than those of mutants lacking only zygotic expression of Ash1 (Shearn, 1989; Shearn et al., 1987). Specifically, *ash1^22 m– z–^* mutant pharate adults showed transformation of the third (T3) to the second thoracic segment (T2) owing to widespread loss of expression of the HOX gene *Ultrabithorax* (*Ubx*) in larval haltere and third-leg imaginal disc primordia that form the T3 segment in adults (Fig. 4B, compare rows 2 and 1). The abdominal segments A5 and A6 of *ash1^22 m– z–^* pharate adults showed transformations towards A4, most noticeable by the loss of pigmentation in males (Fig. 4B, compare rows 2 and 1). Moreover, these males also develop an A7 segment that is normally suppressed in the wild type (Fig. 4B, compare rows 2 and 1). All these transformations are indicative of loss of expression of the HOX gene *Abdominal-B* (*Abd-B*) in the larval primordia of these adult structures. The requirement of Ash1 for normal expression of *Abd-B* is also apparent in the central nervous system (CNS), where *ash1^22 m– z–^* mutant larvae show patchy loss of Abd-B expression (Fig. 4B, compare rows 2 and 1). Animals that completely lack Ash1 protein therefore show a specific HOX loss-of-function syndrome but, perhaps surprisingly, no other obvious morphological defects.

We then investigated whether loss of Ash1 HMTase activity is responsible for these phenotypes by analysing animals that expressed the Ash1^R1464A^ mutant protein instead of wild-type Ash1. As documented above, the R1464A mutation in Ash1 SET domain severely compromises HMTase activity (Fig. 3A). We generated animals that were homozygous for the *ash1^22^* null mutation but carried a single transgene that expressed the Ash1^R1464A^ protein or, as a control, wild-type Ash1 protein from a genomic *ash1* fragment. In the control animals, the transgene-encoded wild-type Ash1 protein fully rescued *ash1^22^* homozygotes into viable and fertile adults that were morphologically indistinguishable from wild-type *Drosophila* and could be maintained as a healthy strain (Fig. 4A). In contrast, the Ash1^R1464A^ mutant protein largely failed to rescue the homeotic phenotypes of *ash1^22^* homozygotes but a substantial fraction of these animals nevertheless eclosed from the pupal case (Fig. 4A). The eclosed first generation, referred to as *ash1^R1464A m+ z–^* mutants, were fertile and this permitted the generation of *ash1^R1464A m– z–^* animals in which not only zygotically expressed but also the maternally supplied Ash1 protein contained the R1464A point mutation. These *ash1^R1464A m– z–^* mutant animals showed loss of HOX gene expression and homeotic transformations almost as severe as *ash1^22 m– z–^* mutant animals (Fig. 4B, compare rows 3 and 2). Moreover, the *ash1^R1464A m– z–^* mutant animals that eclosed from the pupal case invariably died after 1-2 days (Fig. 4A). The inability of the Ash1^R1464A^ protein to maintain normal HOX gene expression indicates that Ash1 HMTase activity is crucial for this process. The slightly less severe phenotype of *ash1^R1464A m– z–^* mutants and the finding that a fraction of these animals even eclose from the pupal case could be explained by low levels of residual HMTase activity of the Ash1^R1464A^ protein *in vivo* or, alternatively, by an HMTase-independent function of Ash1 in maintaining HOX gene transcription.

Finally, we tested the requirement of MRG15 for AMC function *in vivo*. To analyse *Drosophila* mutants lacking the MRG15 protein, we used homologous recombination (Gong and Golic, 2003) to generate *MRG15*^Δ^, an allele that deletes almost the entire MRG15-coding region (Fig. S2). Among the animals homozygous for *MRG15*^Δ^ that were derived from heterozygous parents, a substantial fraction developed into adults that eclosed from the pupal case (Fig. 4A) and showed mild HOX loss-of-function phenotypes in the adult epidermis as their only detectable morphological defect. Many of these *MRG15*^Δ^ *^m+ z–^* animals died shortly after eclosing; better survival was observed in animals that were trans-heterozygous for *MRG15*^Δ^ and *Df(3R)BSC741*, another chromosomal deletion that removes the entire *MRG15* gene and additional flanking genes. For all experiments described below, we therefore used *MRG15*^Δ^*/Df(3R)BSC741* trans-heterozygous animals but for simplicity refer to them as *MRG15*^Δ^ mutants. *MRG15*^Δ^ *^m+ z–^* surviving adults were fertile and produced *MRG15*^Δ^ *^m–^* ^*z*–^ progeny that lacked both maternally-deposited and zygotically-expressed MRG15 protein. A fraction of these *MRG15*^Δ^ *^m–^* ^*z*–^ mutant animals again developed into pupae and adults (Fig. 4A) that, intriguingly, showed loss of HOX gene expression and homeotic transformations that overall were almost as severe as those observed in *ash1^R1464A m– z–^* mutants (Fig. 4B, compare rows 4 and 3). This striking similarity of the *MRG15* null and *ash1* catalytic mutant phenotypes, together with the finding that MRG15 is required for efficient H3K36 di-methylation by Ash1 *in vitro* (Fig. 3), implies that MRG15 is also important for Ash1 HMTase activity *in vivo*. We note that the survival of *MRG15*^Δ^ *^m– z–^* mutant animals to adulthood and the specific homeotic phenotypes may seem surprising because MRG15 is also present in other chromatin protein complexes, such as the Tip60 complex (Kusch et al., 2004). It therefore appears that the role of MRG15 in AMC likely is the primary vital function of this protein in *Drosophila*. Finally, because of the specific homeotic phenotype of MRG15 mutants, we propose that MRG15 should, like Ash1, be classified as a trxG protein.

### AMC is required for the regulation of several hundred genes

**Fig. 5.**
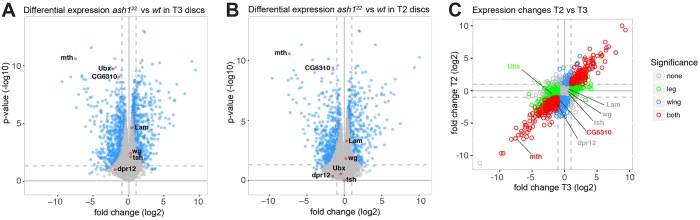
**Alteration of gene expression in *ash1* mutants.** (A,B) Volcano plots of changes in gene expression in *ash1^22^* ^*m+ z−*^ (*ash1^−/−^*) larvae compared with wild-type larvae in T3 discs (A) and in T2 discs (B). Genes selected for the analyses documented in Fig. 6 are labelled. (C) Scatter plot comparing T2 and T3 by log2 fold change of gene expression in *ash1^22^* ^*m+ z−*^ versus wild-type larvae, colour-coded by statistical significance in T2 or T3, or both (*P*<0.01, log2 fold change ≥2). There is high similarity in T3 and T2 tissues. Genes also labelled in A and B are indicated.

The analyses described above provide strong evidence that AMC activity is crucial for maintaining normal expression of HOX genes. To obtain a comprehensive overview of the genes that are regulated by this complex, we compared the transcriptome in imaginal disc tissues from *ash1^22^* homozygous larvae (*ash1^22 m+ z–^*) with that in the same tissues from wild-type larvae. To achieve this, we extracted RNA from hand-dissected batches of haltere and third-leg imaginal discs from the T3 segment, in the following referred to as T3 discs, and, in parallel, also from batches of wing imaginal discs from the T2 segment, referred to as T2 discs, from both wild-type and *ash1^22^* homozygous larvae. Transcriptome sequencing was then performed on at least four independent biological replicates of each type of sample. Bioinformatic analyses of RNA-seq data from *ash1^22^* mutant and wild-type larvae identified several hundred genes that were differentially expressed in both T3 and T2 discs. Specifically, about 300 genes are differentially expressed with a log2 fold change ≥2 and about 600 genes are differentially expressed with a log2 fold change ≥1 (Fig. 5A,B; Table S3). As expected from the analyses shown above (Fig. 4B), *Ubx* was downregulated more than fourfold in *ash1* mutant T3 discs (Fig. 5A) but was not detected as a differentially regulated gene in T2 discs (Fig. 5B) because it is not expressed in that tissue. Importantly, most other genes that were differentially expressed in T3 discs of *ash1* mutant and wild-type larvae were also differentially expressed in T2 discs (Fig. 5C). The altered expression of these genes in *ash1* mutants is therefore not a consequence of reduced expression of the transcription factor Ubx in T3 discs. In summary, these results show that Ash1 is required for the normal expression of a few hundred genes in addition to regulating the HOX genes.

**Fig. 6.**
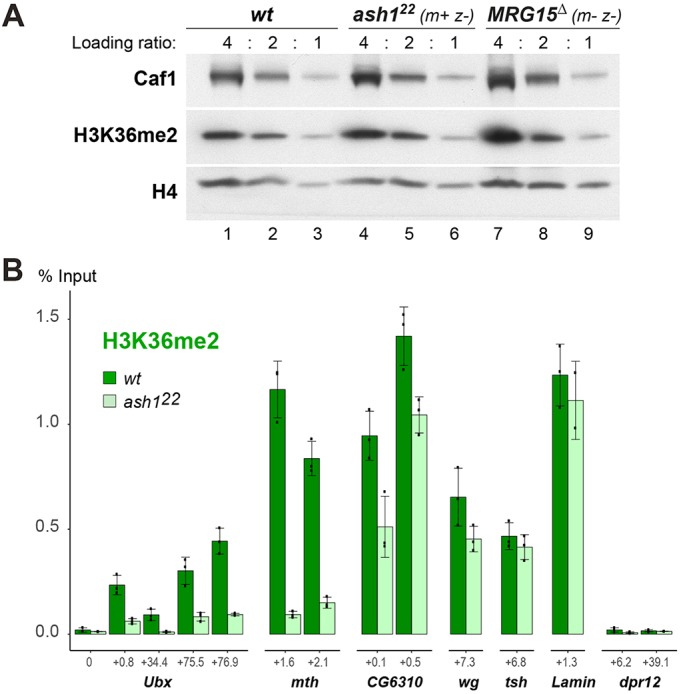
**Ash1 is required for normal H3K36me2 levels at HOX and other target genes.** (A) H3K36me2 bulk levels are unchanged in *ash1* or *MRG15* null mutants. Western blot on serial dilutions (4:2:1) of total extracts from imaginal discs from wild-type (wt), *ash1^22^* ^*m+ z−*^ and *MRG15*^Δ*m– z–*^ mutant larvae, probed with antibodies against H3K36me2 and, as loading control, histone H4 and Caf1. The genotype of the *MRG15*^Δ*m− z−*^ animals is *MRG15*^Δ^*/Df(3R)BSC741* (see text). (B) ChIP qPCR analysis in wild-type (dark-green bars) and in *ash1^22^* homozygous (*ash1^22^*, light-green bars) larvae, monitoring H3K36me2 levels at the *Ubx*, *mth*, *CG6310*, *wg*, *tsh*, *lam* and *dpr12* genes in T3 discs. At each gene, H3K36me2 was analysed at one or more regions, and for each qPCR, amplicon coordinates are indicated as distance in kb from the transcription start site, see also Table S5. For both genotypes, bars show ChIP signals from three independent ChIP reactions that were performed on three independently prepared batches of chromatin and are presented as a percentage of input chromatin precipitated at each region; dots show individual experimental results and error bars show standard deviation.

These results raised the issue of whether Ash1 directly binds to these deregulated genes and whether Ash1 is required for dimethylation of H3K36 in their chromatin. Three separate studies have generated genome-wide Ash1 protein-binding profiles in different *Drosophila* tissue culture cell lines (Huang et al., 2017; Kockmann et al., 2013; Schwartz et al., 2010). Kockmann et al. reported that Ash1 binds in a sharply localized manner in the promoter region of most active genes in the cells they analysed (Kockmann et al., 2013), whereas Schwartz et al. reported that Ash1 is bound at about 50 genomic regions where it is associated with large chromatin domains that span on average about 10 kb (Schwartz et al., 2010); Huang et al. identified around 500 Ash1-bound genes (Huang et al., 2017). To analyse Ash1 binding in the same tissues that we had used for our transcriptome analyses, we attempted to perform ChIP-seq experiments in imaginal discs. However, we failed to enrich sufficient amounts of chromatin by immunoprecipitation with Ash1 antibodies and were therefore unable to generate reliable Ash1 protein-binding profiles. In the following, we therefore focused our analysis on investigating how H3K36me2 levels are affected in mutants lacking AMC.

### Bulk H3K36me2 levels are not diminished in mutants lacking AMC

In the first experiment, we assessed the contribution of AMC to total H3K36me2 levels in developing larvae. We analysed H3K36me2 bulk levels in imaginal disc tissues dissected from wild-type, *ash1^22 m+ z–^* or *MRG15*^Δ^ *^m– z–^* third instar larvae. As shown in Fig. 6A, H3K36me2 bulk levels were not detectably diminished in either of the two mutants. These results suggested that AMC might contribute to H3K36 di-methylation in a more gene-specific manner.

### Ash1 is required for high-level H3K36 di-methylation at Ash1-regulated genes

We next analysed H3K36me2 levels at genes that we had found to be downregulated in *ash1* mutants. We performed chromatin immunoprecipitation (ChIP) assays with H3K36me2 antibodies on chromatin prepared from haltere and third leg imaginal discs (T3 discs) dissected from *ash1^22^* homozygous larvae (*ash1^22 m+ z–^*) or from wild-type larvae and used real-time quantitative PCR (qPCR) to monitor H3K36me2 levels in the transcribed region of specific genes. These genes were: *methuselah* (*mth*), as an example of a gene that is strongly (almost 200-fold) downregulated in *ash1* mutants; *Ubx*, as a moderately (more than fourfold) downregulated gene; and *CG6310*, as a weakly (about twofold) downregulated gene (Fig. 5A). As control, we analysed H3K36me2 at the *wingless* (*wg*), *teashirt* (*tsh*) and *lamin* (*lam*) genes that are all expressed in T3 discs but are not downregulated in *ash1* mutants (Fig. 5A, Table S3). As an additional control, we analysed H3K36me2 at *dpr12*, a gene that is virtually inactive in T3 and T2 discs (Table S3). As illustrated in Fig. 6B, in wild-type animals, H3K36me2 is detected in the coding region of the expressed *Ubx*, *mth*, *CG6310*, *wg*, *tsh* and *lam* genes, but not at the inactive *dpr12* gene (Fig. 6B). In *ash1* mutants, H3K36me2 levels were strongly diminished at *Ubx* and *mth*, and were mildly reduced at *CG6310*, but not significantly changed at *wg*, *tsh* and *lam* (Fig. 6B). Nonetheless, even at the *Ubx* and *mth* genes, where H3K36me2 levels are strongly reduced, the modification is not completely abolished. This suggests that the residual H3K36 di-methylation at these genes is generated by other H3K36 HMTases, most likely by NSD and/or Set2. These HMTases are likely also responsible for H3K36me2 at the *wg*, *tsh* and *lam* genes, where the modification appeared undiminished in *ash1* mutants. It is important to keep in mind that even though the extent of H3K36me2 reduction appears to roughly match the extent to which expression of target genes is reduced in *ash1* mutants, the H3K36me2 reduction might not be uniform across the cell population. A case in point for this is *Ubx*, where expression in T3 discs of *ash1* mutants is lost in a mosaic all-or-none fashion (Fig. 4B). It is possible that, in cells showing loss of *Ubx* expression, H3K36me2 might be completely lost from *Ubx* chromatin and that the residual H3K36me2 ChIP signal at *Ubx* in *ash1* mutants (Fig. 6B) represents H3K36me2 at *Ubx* in those cells that retained normal levels of *Ubx* expression. In summary, these analyses show that genes that are downregulated in *ash1* mutants show a reduction, but not complete loss, of H3K36me2 in their chromatin.

## DISCUSSION

Biochemical studies over the past have revealed that almost all PcG and trxG regulators originally identified through genetics are subunits of multi-protein complexes that modify chromatin; this has greatly helped to unravel the molecular mechanism of these proteins and to understand how they function (Kassis et al., 2017). Here, we have investigated the molecular interactions and the mechanism of action of the trxG protein Ash1 and its role in regulating gene expression in *Drosophila*. The work reported in this study leads to the following main conclusions. First, biochemical purifications from *Drosophila* show that Ash1 is the subunit of a multi-protein complex that contains MRG15 and Caf1: the AMC complex. Second, reconstitution of AMC using recombinant proteins uncovered that the MRG domain of MRG15 binds to a conserved FxLP motif next to the SET domain of Ash1, and that this interaction greatly enhances Ash1 catalytic activity for H3K36 di-methylation in nucleosomes. A recent study by Bing Zhu's lab reported the purification of an identical Ash1-MRG15-Caf1 complex and they also found that MRG15 stimulates Ash1 enzymatic activity *in vitro* (Huang et al., 2017), providing independent support for these first two conclusions from our work. Third, our transcriptome analyses in developing *Drosophila* reveal that AMC is required for the normal expression of a few hundred genes in addition to the HOX genes. Fourth, we show that animals that lack Ash1 or MRG15, or contain a catalytically inactive version of Ash1 have the capacity to go through embryonic, larval and pupal development, and complete metamorphosis to differentiate into adults that show very specific homeotic phenotypes as their main morphological defect. Notably, this defines MRG15 as a novel trxG protein. Fifth, we find that AMC does not make a major contribution to the bulk of H3K36 di-methylation in *Drosophila* but that the complex is essential for generating wild-type levels of H3K36me2 at HOX and other target genes that are downregulated in *ash1* mutants. In the following, we shall focus on specific aspects of these findings.

### Activation of Ash1 methyltransferase activity by MRG15

Our analyses and those of Huang et al. (2017) strongly suggest that AMC rather than the Ash1 protein alone is the active form of this HMTase and that the MRG15 subunit stimulates Ash1 HMTase activity via a mechanism that is conserved in flies and mammals. In particular, the data sets in Fig. 3 collectively suggest that interaction of the MRG domain with the FxLP motif preceding the Ash1/ASH1L SET domain increases the catalytic activity of this domain in both the fly and the human complex. Without structural information about this interaction, we can currently only speculate on the activation mechanism. An attractive possibility would be that MRG15 binding allosterically activates the SET domain by displacing the auto-inhibitory loop formed by the Ash1 post-SET domain (An et al., 2011) and thereby facilitates access of the H3K36 substrate lysine to the catalytic centre in the SET domain. In addition to this allosteric activation mechanism, MRG15 might also promote AMC activity through a second mechanism involving interaction of the MRG15 chromo barrel domain with nucleosomes that already carry the H3K36me2 and/or -me3 modification (Zhang et al., 2006). Specifically, in the chromatin of actively transcribed genes that contain H3K36me2/3-modified nucleosomes, interaction of MRG15 with such nucleosomes – perhaps in cooperation with interactions of the Ash1 bromodomain or PHD finger with other histone modifications – might permit AMC to di-methylate H3K36 in unmodified neighbouring nucleosomes more efficiently. This might be particularly crucial when histone H3 is exchanged in the wake of transcription or when newly synthesized octamers containing unmodified H3 are incorporated after DNA replication. In this context, it should also be recalled that the target genes *Ubx*, *mth* or *CG6310* still contain low levels of H3K36me2-modified nucleosomes in discs of *ash1^22 m+ z–^* mutant larvae. This residual H3K36me2 is unlikely to represent nucleosomes that were modified by maternally-deposited Ash1 protein. First, Ash1 protein is undetectable in these cells (Fig. S1) and, second, the replication-coupled dilution of parental nucleosomes in these dividing cells will require *de novo* methylation of newly incorporated H3 molecules at every S-phase. It therefore seems more likely that the low level of H3K36me2 at these genes in *ash1* mutants is generated by NSD and Set2. According to this view, Ash1 would thus act on top of an H3K36me2/3 landscape generated by these more globally acting H3K36-methylating enzymes. Furthermore, Ash1 association with polytene chromosomes was reported to depend on Kismet/CHD7, a nucleosome remodelling factor that is required for the transition from transcription initiation to transcriptional elongation (Srinivasan et al., 2008). Together, these observations all point to a scenario where AMC acts on chromatin that already is transcriptionally active and possibly already is at least partially decorated with H3K36me2/3.

### Developmental and gene expression defects in mutants lacking AMC function

Our transcriptome analyses identified several hundred genes that are de-regulated in *ash1* mutants. This observation may seem surprising given that *ash1*-null and catalytic mutants nevertheless develop into pharate or even viable and fertile adults, respectively. The homeotic transformations in *ash1* mutants show that downregulation of HOX gene expression has a clear physiological consequence. We have not been able to detect any other obvious morphological defects in the epidermal structures of *ash1* mutants. However, it is possible that changes in the expression levels of AMC-regulated genes other than HOX genes cause morphological defects in internal structures or organs, or that they affect the physiology of mutant animals and could in this way impact on their survival and viability. Future studies will be needed to assess the consequences of altered expression levels of the non-HOX genes regulated by AMC. Here, we shall focus our discussion on the role of AMC in regulating HOX genes where lack of the complex and the reduction of H3K36me2 have a clear physiological consequence.

### Requirement for H3K36 dimethylation by AMC to counteract Polycomb repression

As discussed in the Introduction, genetic and molecular studies originally uncovered that Ash1 prevents H3K27 tri-methylation by PRC2 in the coding region of the *Ubx* gene in cells where this gene is expressed (Papp and Müller, 2006). Biochemical studies *in vitro* then established that PRC2 HMTase activity for H3K27 methylation is inhibited on nucleosomes carrying H3K36me2 (Schmitges et al., 2011; Yuan et al., 2011). Here, we now show that Ash1 is indeed required for deposition of normal levels of H3K36me2 in the *Ubx*-coding region in cells where *Ubx* is normally expressed (Fig. 6B). Together, this supports a model in which AMC di-methylates H3K36 in the *Ubx*-coding region and thereby antagonizes H3K27me3 deposition by PRC2 and the instalment of Polycomb repression at this gene. It remains to be investigated whether H3K36 di-methylation by AMC also antagonizes H3K27 tri-methylation at other Ash1-regulated genes. Inspection of the available H3K27me3 profiles in wild-type *Drosophila* embryonic, larval or adult cells, or in different tissue culture cell lines provides no evidence for presence of H3K27me3 at the *mth* or *CG6310* genes in any of these cells (www.modencode.org). At these genes, AMC might therefore preserve normal levels of expression through mechanisms other than counteracting Polycomb repression.

A conspicuous feature of mutants lacking AMC function is the all-or-none loss of HOX gene expression. Specifically, the patchy loss of *Ubx* and *Abd-B* expression and the patchy transformations in the adult epidermis of *ash1*-null, *ash1* catalytic inactive or *MRG15*-null mutants suggest that expression of these genes is lost in a stochastic fashion in a fraction of larval cells and, once lost, this ‘OFF’ state is then clonally propagated in their daughter cells (Fig. 4B). Conversely, in other cells, HOX gene expression appears to be maintained (Fig. 4B). How could this variegated loss of expression be explained? As discussed above, the low levels of H3K36me2 at *Ubx* in *ash1* mutants are likely generated by NSD and/or Set2. It is possible that, in the absence of AMC, H3K36 methylation by these other HMTases may suffice to sustain H3K36me2 levels on at least one of the two *Ubx* alleles on the two homologous chromosomes above the threshold level needed to antagonize PRC2. We imagine that this crucial threshold is, however, not reliably reached in all cells, and, as a consequence, gain of H3K27 tri-methylation by PRC2 on both *Ubx* alleles may result in a stable OFF switch that is then clonally propagated. According to this view, a key physiological role of the trxG protein complex AMC is to augment the number of H3K36me2-modified nucleosomes across the chromatin of active HOX genes to safeguard them from H3K27 tri-methylation by PRC2.

## MATERIALS AND METHODS

### *Drosophila* strains

Strains with the following genotypes were generated and/or used in this study:

*w* (wild type);

*w; NTAP-Ash1; ash1^22^ FRT2A*;

*w; ash1-CTAP/CyO; ash1^22^ FRT2A*;

*w; ash1^22^ FRT2A/TM3 twi::EGFP*;

*w hsp70-flp; ovo^D^ FRT2A/TM2/TM6B*;

*w; ash1^wt^(VK37); ash1^22^ FRT2A*;

*w; ash1^R1464A^ (VK37); ash1^22^ FRT2A/TM6B*;

*w; MRG15*^Δ^/*TM6C*; and *w; Df(3R)BSC741/TM3 twi::EGFP*.

The genotypes of the animals shown in Fig. 4 were as follows: *ash1^22 m− z−^* animals were *w; ash1^22^ FRT2A* and derived from crossing *w; ash1^22^ FRT2A/ovo^D^ FRT2A* females (with germ line clones) with *w; ash1^22^ FRT2A/ TM3 twi::EGFP* males; *ash1^R1464A m− z−^* animals were *w; ash1^R1464A^ (VK37)/+; ash1^22^ FRT2A* and were derived from crossing *w; ash1^R1464A^ (VK37); ash1^22^ FRT2A* females with *w; ash1^22^ FRT2A/TM3 twi::EGFP* males; and *MRG15*^Δ *m− z*^^−^ animals were *w; MRG15*^Δ^*/Df(3R)BSC741* and derived from crossing *w; MRG15*^Δ^*/Df(3R)BSC741* females with *w; Df(3R)BSC741*/*twi::EGFP* males.

### Antibodies

Antibodies used in this study are listed in Table S4. Antibodies against Ash1_517-842_ were raised in rabbits. The Ash1_517-842_ epitope was expressed as a 6×His-tagged protein in *Escherichia coli* and purified under denaturing conditions; the same epitope was used for affinity purification of the antibody.

### Tandem affinity purification (TAP) of Ash1 complexes

The *NTAP-Ash1* and *Ash1-CTAP* transgenes both contained the entire Ash1_1-2226_ open reading frame in the previously described *Drosophila* transformation vectors CaSpeR-NTAP and CaSper-CTAP, respectively (Klymenko et al., 2006); plasmid maps are available on request. Tandem affinity purifications were performed from embryonic nuclear extracts of *w; NTAP-Ash1; ash1^22^ FRT2A* and *w; ash1-CTAP/CyO; ash1^22^ FRT2A* strains as described previously (Klymenko et al., 2006).

### Mass spectrometric analysis of proteins isolated by TAP

Eluates from calmodulin beads after TAP were separated on 4-12% polyacrylamide gels. One part of the material was used for silver staining to visualize the proteins for illustration (Fig. 1A). For mass spectrometric analysis, the bulk part of the material was separated on the same type of gel, the entire gel lane was excised and subdivided into different slices that were then each alkylated and digested with trypsin as described (Barth et al., 2014). Peptides were collected by acid extraction, concentrated by evaporation and resuspended in 0.1% TFA. Fifty percent of the digested material was injected into an Ultimate 3000 HPLC system (Thermo-Fisher Scientific) and analysed as described previously (Barth et al., 2014). For protein identification, the raw data were analysed with the Andromeda algorithm of the MaxQuant protein analysis package (version 1.5.3.30) against the FlyBase dmel-all-translation-r5.32.fasta database, including reverse sequences and contaminants. For quantification, Intensity Based Absolute Quantification (iBAQ) values were calculated from peptide intensities. For presentation, values were log2-transformed and subsequently missing values were imputed from a random distribution centred at 1/3×log2 of the obtained experimental data (Table S1). Ash1, MRG15 and Caf1 peptides identified by mass spectrometry are listed in Table S2.

### Expression and purification of recombinant proteins

*Drosophila* Ash1_C_, and the complete coding sequences of MRG15, Msl3, Caf1 and Esc were cloned into pFastBac1 (ThermoFisher) with appropriate affinity-tag coding sequences at their N termini: StrepII-MRG15, StrepII-Msl3, StrepII-Caf1, StrepII-Esc, His6-Ash1_C_, His6-Ash1_C_^RxRP^ and His6-Ash1_C_^R1464A^. Plasmids and viruses are available on request. Fig. 2B-D and Fig. 3 show Strep-Tactin affinity purifications. Insect cells were lysed in Strep-Buffer A [20 mM Tris-HCl (pH 8), 300 mM KCl, 2 mM MgCl_2_, 15% glycerol, 10 µM ZnSO_4_, 0.1% NP-40 substitute, 1 mM 1,4-dithiothreitol and protease inhibitors]. Cleared lysates were loaded on Strep-Tactin sepharose beads (IBA) and washed multiple times with Strep-Buffer A. Retained proteins were released in Strep-Buffer B [20 mM Tris-HCl (pH 8), 150 mM NaCl, 10% glycerol, 0.5 mM 1,4-dithiothreitol and protease inhibitors] supplemented with 5 mM d-desthiobiotin (Sigma). For affinity purification using the His-affinity tag in Fig. 2E, cells were lysed in His-buffer A [20 mM Tris-HCl (pH 8), 300 mM KCl, 4 mM MgCl_2_, 5 mM imidazole, 5% glycerol, 10 µM ZnSO_4_, 0.05% NP-40 substitute, 4 mM β-mercaptoethanol and protease inhibitors] and subjected to a Ni-affinity purification (Ni-NTA Agarose, Qiagen). After multiple washes with His-buffer A, proteins were eluted with His-Buffer B [20 mM Tris-HCl (pH 8), 300 mM NaCl, 250 mM imidazole, 5% glycerol, 0.05% NP-40 substitute, 4 mM β-mercaptoethanol and protease inhibitors].

For expression in *E. coli*, *Drosophila* Ash1_XL-SET_ and human ASH1L_XL-SET_ were cloned into a modified pET28a-TEV vector, containing an N-terminal 6×His-tag and a TEV protease site. *Drosophila* full-length MRG15 and human MRG15_MRG_ domain were cloned into pET21a vector. Ash1_XL-SET_ and ASH1L_XL-SET_ proteins were expressed and Ash1_XL-SET_:MRG15 and ASH1L_XL-SET_:MRG15_MRG_ complexes were co-expressed in *E. coli* BL21 (RILP) cells. The proteins were then purified on Ni-NTA (Qiagen) resin. Following treatment with TEV protease to remove the N-terminal 6×His-tag, the proteins were further purified by ion exchange, and size exclusion chromatography in a buffer containing 50 mM Tris HCl (pH 8.0) and 100 mM NaCl.

### HMTase assays

Mononucleosome substrates were reconstituted with recombinant *Drosophila* histones and a 215 bp long 601 DNA fragment. HMTase reactions on mononucleosomes were performed in buffer containing 80 µM SAM, 65 mM Tris-HCl (pH 8.5), 78 mM NaCl, 2.5 mM MgCl_2_, 0.23 mM EDTA (pH 8.0), 1 mM DTT, 1 mM β-mercaptoethanol, 2.6 mM d-desthiobiotin, 5% glycerol and protease inhibitors, and were incubated for 3 h at 25°C. For HMTase reactions on oligonucleosomes, nucleosomal arrays were assembled with recombinant *Xenopus* histones and G5E4 DNA. Reactions were performed in buffer containing 2 µM ^3^H-SAM, 50 mM Tris-HCl (pH 9.0), 40 mM NaCl, 5 mM MgCl2 and 4 mM DTT, and were incubated for 80 min at 37°C. The reactions were analysed on a 15% SDS-polyacrylamide gel, visualized by Coomassie Blue staining or transferred to an immobilon-PSQ PVDF membrane (ISEQ00010, Millipore) and exposed to an image plate. The image plate was scanned with FLA-7000 (Fuji Film) for autoradiography.

### Ash1 genomic transgenes

Transgenes containing genomic *ash1* fragments comprised BDGP R6.14 chr3L sequences 19,600,040…19,590,604, using BAC CH322-147P9 as template. In the *Taf6*-coding sequence present in this fragment, multiple ATG initiation codons were converted into stop codons. For the *ash1^R1464A^* transgene, the AGG codon for Arg_1464_ was mutated to GCG. The genomic fragments were cloned into a modified attB vector (pUMR-FLAP) and integrated at the attP site VK37 (BDSC 9752).

### Generation of the *MRG15^*Δ*^* deletion allele

*MRG15^*Δ*^* was generated by replacing BDGP R6.14 chr3R: 15,276,676…15,277,889 with *miniwhite* marker gene using ends-out targeting with the pw35 vector (Gong and Golic, 2003). The 5′ homology arm (BDGP R6.14)chr3R:15,277,890…15,281,975) and the 3′ homology arm (BDGP R6.14 chr3R:15,272,003…15,276,675) shown in Fig. S2 were amplified from BAC CH322-160G6 and the initiation ATG of *MRG15* was mutated to ATC. The *MRG15*^Δ^ allele isolated and used throughout this study was selected among multiple independent targeting events after confirming that *MRG15* was disrupted (Fig. S2) and that sequences in the homology arms and flanking DNA were unaltered.

### Immunostaining and adult cuticle preparations of *Drosophila*

Immunostaining of imaginal discs and preparations of adult cuticles were performed following standard protocols (Beuchle et al., 2001).

### Western blot analyses on larval tissue extracts

Western blots were performed on total extracts prepared from pooled hand-dissected wing, haltere and 3rd leg imaginal discs of a given genotype (Copur and Müller, 2013).

### Transcriptome analysis by NGS

RNA was isolated from independently prepared batches of hand-dissected haltere and third-leg imaginal discs (T3 discs; discs from eight larvae for each biological replicate) or wing discs (T2 discs; discs from eight larvae for each biological replicate) from wild-type or *ash1^22^* homozygous larvae, using the Direct-zol RNA mini Prep Kit (Zymo Research). After additional Agencourt AMPure XP purification (Beckman Coulter), isolated RNA was analysed on a Bioanalyzer (Agilent) using an RNA 6000 Nano Chip (Agilent). Non-degraded RNA from at least four independent biological replicates of each type of sample was used to construct sequencing libraries using sense mRNA-Seq Library Prep Kit (Lexogen). Quality-controlled and quantified libraries were sequenced on an HiSeq1500 system (Illumina) in the single-end mode (100 nt read length).

For analysis, trimmed and quality-filtered reads were mapped using the STAR aligner (Dobin et al., 2013) to the Ensembl genome annotation and *Drosophila* genome assembly dm3. Read counts were quantified using featurecounts (Liao et al., 2014) and differential gene expression calculated with limma using the voom transformation (Law et al., 2014).

### ChIP analysis

Chromatin preparation from hand-dissected haltere and third-leg imaginal discs (T3 discs) from wild-type or *ash1^22^* homozygous larvae and ChIP analysis was performed as described (Laprell et al., 2017). For each biological replicate, chromatin prepared from the discs of 60 larvae were used as input material. ChIP was performed with polyclonal anti-H3K36me2 antibody (Abcam ab9049) and qPCR primers used for analysis are listed in Table S5.

## Supplementary Material

Supplementary information

Supplementary information

## References

[DEV163808C1] An S., Yeo K. J., Jeon Y. H. and Song J.-J. (2011). Crystal structure of the human histone methyltransferase ASH1L catalytic domain and its implications for the regulatory mechanism. 286, 8369-8374. 10.1074/jbc.M110.203380 PMC3048721 21239497

[DEV163808C2] Anderson A. E., Karandikar U. C., Pepple K. L., Chen Z., Bergmann A. and Mardon G. (2011). The enhancer of trithorax and polycomb gene Caf1/p55 is essential for cell survival and patterning in Drosophila development. 138, 1957-1966. 10.1242/dev.058461 PMC3082301 21490066

[DEV163808C3] Barth T. K., Schade G. O. M., Schmidt A., Vetter I., Wirth M., Heun P., Thomae A. W. and Imhof A. (2014). Identification of novel Drosophila centromere-associated proteins. 14, 2167-2178. 10.1002/pmic.201400052 24841622

[DEV163808C4] Bell O., Wirbelauer C., Hild M., Scharf A. N. D., Schwaiger M., MacAlpine D. M., Zilbermann F., van Leeuwen F., Bell S. P., Imhof A. et al. (2007). Localized H3K36 methylation states define histone H4K16 acetylation during transcriptional elongation in Drosophila. 26, 4974-4984. 10.1038/sj.emboj.7601926 PMC2140113 18007591

[DEV163808C50] Beuchle D., Struhl G. and Müller J. (2001). Polycomb group proteins and heritable silencing of Drosophila Hox genes. 128, 993-1004. 10.1242/dev.128.6.993 11222153

[DEV163808C5] Bowman S. K., Deaton A. M., Domingues H., Wang P. I., Sadreyev R. I., Kingston R. E. and Bender W. (2014). H3K27 modifications define segmental regulatory domains in the Drosophila bithorax complex. 3, e02833 10.7554/eLife.02833 PMC4139060 25082344

[DEV163808C51] Copur Ö. and Müller J. (2013). The histone H3-K27 demethylase Utx regulates HOX gene expression in *Drosophila* in a temporally restricted manner. 140, 3478-3485. 10.1242/dev.097204 PMC3912917 23900545

[DEV163808C6] Dobin A., Davis C. A., Schlesinger F., Drenkow J., Zaleski C., Jha S., Batut P., Chaisson M. and Gingeras T. R. (2013). STAR: ultrafast universal RNA-seq aligner. 29, 15-21. 10.1093/bioinformatics/bts635 PMC3530905 23104886

[DEV163808C7] Dorighi K. M. and Tamkun J. W. (2013). The trithorax group proteins Kismet and ASH1 promote H3K36 dimethylation to counteract Polycomb group repression in Drosophila. 140, 4182-4192. 10.1242/dev.095786 PMC3787758 24004944

[DEV163808C8] Eram M. S., Kuznetsova E., Li F., Lima-Fernandes E., Kennedy S., Chau I., Arrowsmith C. H., Schapira M. and Vedadi M. (2015). Kinetic characterization of human histone H3 lysine 36 methyltransferases, ASH1L and SETD2. 1850, 1842-1848. 10.1016/j.bbagen.2015.05.013 26002201

[DEV163808C52] Gambetta M. C., Oktaba K. and Müller J. (2009). Essential role of the glycosyltransferase Sxc/Ogt in Polycomb repression. 325, 93-96. 10.1126/science.1169727 19478141

[DEV163808C9] Gaydos L. J., Rechtsteiner A., Egelhofer T. A., Carroll C. R. and Strome S. (2012). Antagonism between MES-4 and Polycomb repressive complex 2 promotes appropriate gene expression in C. elegans germ cells. 2, 1169-1177. 10.1016/j.celrep.2012.09.019 PMC3513488 23103171

[DEV163808C10] Gong W. J. and Golic K. G. (2003). Ends-out, or replacement, gene targeting in Drosophila. 100, 2556-2561. 10.1073/pnas.0535280100 PMC151379 12589026

[DEV163808C11] Huang C., Yang F., Zhang Z., Zhang J., Cai G., Li L., Zheng Y., Chen S., Xi R. and Zhu B. (2017). Mrg15 stimulates Ash1 H3K36 methyltransferase activity and facilitates Ash1 Trithorax group protein function in Drosophila. 8, 1649 10.1038/s41467-017-01897-3 PMC5696344 29158494

[DEV163808C12] Kang H., Jung Y. L., McElroy K. A., Zee B. M., Wallace H. A., Woolnough J. L., Park P. J. and Kuroda M. I. (2017). Bivalent complexes of PRC1 with orthologs of BRD4 and MOZ/MORF target developmental genes in Drosophila. 31, 1988-2002. 10.1101/gad.305987.117 PMC5710143 29070704

[DEV163808C53] Kassis J. A., Kennison J. A. and Tamkun J. W. (2017). Polycomb and trithorax group genes in Drosophila. 206, 1699-1725. 10.1534/genetics.115.185116 PMC5560782 28778878

[DEV163808C13] Klymenko T. and Müller J. (2004). The histone methyltransferases Trithorax and Ash1 prevent transcriptional silencing by Polycomb group proteins. 5, 373-377. 10.1038/sj.embor.7400111 PMC1299022 15031712

[DEV163808C14] Klymenko T., Papp B., Fischle W., Köcher T., Schelder M., Fritsch C., Wild B., Wilm M. and Müller J. (2006). A Polycomb group protein complex with sequence-specific DNA-binding and selective methyl-lysine-binding activities. 20, 1110-1122. 10.1101/gad.377406 PMC1472471 16618800

[DEV163808C15] Kockmann T., Gerstung M., Schlumpf T., Xhinzhou Z., Hess D., Beerenwinkel N., Beisel C. and Paro R. (2013). The BET protein FSH functionally interacts with ASH1 to orchestrate global gene activity in Drosophila. 14, R18 10.1186/gb-2013-14-2-r18 PMC4053998 23442797

[DEV163808C16] Krogan N. J., Kim M., Tong A., Golshani A., Cagney G., Canadien V., Richards D. P., Beattie B. K., Emili A., Boone C. et al. (2003). Methylation of histone H3 by Set2 in Saccharomyces cerevisiae is linked to transcriptional elongation by RNA polymerase II. 23, 4207-4218. 10.1128/MCB.23.12.4207-4218.2003 PMC427527 12773564

[DEV163808C17] Kusch T., Florens L., Macdonald W. H., Swanson S. K., Glaser R. L., Yates J. R., Abmayr S. M., Washburn M. P. and Workman J. L. (2004). Acetylation by Tip60 is required for selective histone variant exchange at DNA lesions. 306, 2084-2087. 10.1126/science.1103455 15528408

[DEV163808C18] Kwong C., Adryan B., Bell I., Meadows L., Russell S., Manak J. R. and White R. (2008). Stability and dynamics of polycomb target sites in Drosophila development. 4, e1000178 10.1371/journal.pgen.1000178 PMC2525605 18773083

[DEV163808C19] LaJeunesse D. and Shearn A. (1995). Trans-regulation of thoracic homeotic selector genes of the Antennapedia and bithorax complexes by the trithorax group genes: absent, small, and homeotic discs 1 and 2. 53, 123-139. 10.1016/0925-4773(95)00430-0 8555105

[DEV163808C20] Langlais K. K., Brown J. L. and Kassis J. A. (2012). Polycomb group proteins bind an engrailed PRE in both the “ON” and “OFF” transcriptional states of engrailed. 7, e48765 10.1371/journal.pone.0048765 PMC3490902 23139817

[DEV163808C21] Laprell F., Finkl K. and Müller J. (2017). Propagation of Polycomb-repressed chromatin requires sequence-specific recruitment to DNA. 356, 85-88. 10.1126/science.aai8266 28302792

[DEV163808C22] Larschan E., Alekseyenko A. A., Gortchakov A. A., Peng S., Li B., Yang P., Workman J. L., Park P. J. and Kuroda M. I. (2007). MSL complex is attracted to genes marked by H3K36 trimethylation using a sequence-independent mechanism. 28, 121-133. 10.1016/j.molcel.2007.08.011 17936709

[DEV163808C23] Law C. W., Chen Y., Shi W. and Smyth G. K. (2014). voom: precision weights unlock linear model analysis tools for RNA-seq read counts. 15, R29 10.1186/gb-2014-15-2-r29 PMC4053721 24485249

[DEV163808C24] Liao Y., Smyth G. K. and Shi W. (2014). featureCounts: an efficient general purpose program for assigning sequence reads to genomic features. 30, 923-930. 10.1093/bioinformatics/btt656 24227677

[DEV163808C25] Miyazaki H., Higashimoto K., Yada Y., Endo T. A., Sharif J., Komori T., Matsuda M., Koseki Y., Nakayama M., Soejima H. et al. (2013). Ash1l methylates Lys36 of histone H3 independently of transcriptional elongation to counteract polycomb silencing. 9, e1003897 10.1371/journal.pgen.1003897 PMC3820749 24244179

[DEV163808C26] Papp B. and Müller J. (2006). Histone trimethylation and the maintenance of transcriptional ON and OFF states by trxG and PcG proteins. 20, 2041-2054. 10.1101/gad.388706 PMC1536056 16882982

[DEV163808C27] Pokholok D. K., Harbison C. T., Levine S., Cole M., Hannett N. M., Lee T. I., Bell G. W., Walker K., Rolfe P. A., Herbolsheimer E. et al. (2005). Genome-wide map of nucleosome acetylation and methylation in yeast. 122, 517-527. 10.1016/j.cell.2005.06.026 16122420

[DEV163808C28] Rigaut G., Shevchenko A., Rutz B., Wilm M., Mann M. and Séraphin B. (1999). A generic protein purification method for protein complex characterization and proteome exploration. 17, 1030-1032. 10.1038/13732 10504710

[DEV163808C29] Schmitges F. W., Prusty A. B., Faty M., Stützer A., Lingaraju G. M., Aiwazian J., Sack R., Hess D., Li L., Zhou S. et al. (2011). Histone methylation by PRC2 is inhibited by active chromatin marks. 42, 330-341. 10.1016/j.molcel.2011.03.025 21549310

[DEV163808C30] Schwartz Y. B., Kahn T. G., Stenberg P., Ohno K., Bourgon R. and Pirrotta V. (2010). Alternative epigenetic chromatin states of polycomb target genes. 6, e1000805 10.1371/journal.pgen.1000805 PMC2799325 20062800

[DEV163808C31] Shearn A. (1989). The ash-1, ash-2 and trithorax genes of Drosophila melanogaster are functionally related. 121, 517-525. 10.1093/genetics/121.3.517 PMC1203637 2497049

[DEV163808C32] Shearn A., Hersperger E. and Hersperger G. (1987). Genetic studies of mutations at two loci of Drosophila melanogaster which cause a wide variety of homeotic transformations. 196, 231-242. 10.1007/BF00376347 28305698

[DEV163808C33] Srinivasan S., Dorighi K. M. and Tamkun J. W. (2008). Drosophila Kismet regulates histone H3 lysine 27 methylation and early elongation by RNA polymerase II. 4, e1000217 10.1371/journal.pgen.1000217 PMC2563034 18846226

[DEV163808C34] Sun B., Hong J., Zhang P., Dong X., Shen X., Lin D. and Ding J. (2008). Molecular basis of the interaction of Saccharomyces cerevisiae Eaf3 chromo domain with methylated H3K36. 283, 36504-36512. 10.1074/jbc.M806564200 PMC2662307 18984594

[DEV163808C35] Tanaka Y., Katagiri Z.-I., Kawahashi K., Kioussis D. and Kitajima S. (2007). Trithorax-group protein ASH1 methylates histone H3 lysine 36. 397, 161-168. 10.1016/j.gene.2007.04.027 17544230

[DEV163808C36] Tripoulas N., LaJeunesse D., Gildea J. and Shearn A. (1996). The Drosophila ash1 gene product, which is localized at specific sites on polytene chromosomes, contains a SET domain and a PHD finger. 143, 913-928. 10.1093/genetics/143.2.913 PMC1207348 8725238

[DEV163808C37] Venkatesh S. and Workman J. L. (2013). Set2 mediated H3 lysine 36 methylation: regulation of transcription elongation and implications in organismal development. 2, 685-700. 10.1002/wdev.109 PMC3767924 24014454

[DEV163808C38] Xiao T., Hall H., Kizer K. O., Shibata Y., Hall M. C., Borchers C. H. and Strahl B. D. (2003). Phosphorylation of RNA polymerase II CTD regulates H3 methylation in yeast. 17, 654-663. 10.1101/gad.1055503 PMC196010 12629047

[DEV163808C39] Xie T., Graveline R., Kumar G. S., Zhang Y., Krishnan A., David G. and Radhakrishnan I. (2012). Structural basis for molecular interactions involving MRG domains: implications in chromatin biology. 20, 151-160. 10.1016/j.str.2011.10.019 PMC3259534 22244764

[DEV163808C40] Xie T., Zmyslowski A. M., Zhang Y. and Radhakrishnan I. (2015). Structural Basis for Multi-specificity of MRG Domains. 23, 1049-1057. 10.1016/j.str.2015.03.020 PMC4456287 25960410

[DEV163808C41] Xu C., Cui G., Botuyan M. V. and Mer G. (2008). Structural basis for the recognition of methylated histone H3K36 by the Eaf3 subunit of histone deacetylase complex Rpd3S. 16, 1740-1750. 10.1016/j.str.2008.08.008 PMC2582589 18818090

[DEV163808C42] Yuan W., Xu M., Huang C., Liu N., Chen S. and Zhu B. (2011). H3K36 methylation antagonizes PRC2-mediated H3K27 methylation. 286, 7983-7989. 10.1074/jbc.M110.194027 PMC3048685 21239496

[DEV163808C43] Zhang P., Du J., Sun B., Dong X., Xu G., Zhou J., Huang Q., Liu Q., Hao Q. and Ding J. (2006). Structure of human MRG15 chromo domain and its binding to Lys36-methylated histone H3. 34, 6621-6628. 10.1093/nar/gkl989 PMC1747190 17135209

