# Supplementary material for: Regulation and function of H3K36 di-methylation by the trithorax-group protein complex AMC
Source: Development. 2018 Apr 5;145(7):dev163808. doi: 10.1242/dev.163808 (PMC5963871; doi:10.1242/dev.163808)
Supplement: Supplementary information [file develop-145-163808-s1.pdf]

SUPPLEMENTAL FIGURES

Figure S1

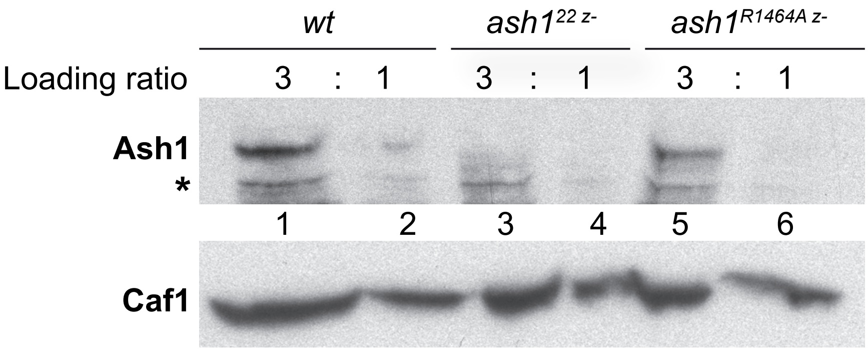

Figure S1

ash1^{22} mutants lack Ash1 protein and the Ash1^{R1464A} protein is expressed at levels similar to wild-type Ash1. Western Blot on total cell extracts from brains and imaginal discs of *wildtype (wt)*, *ash1*$^{22\ z-}$ or *ash1*$^{R1464A\ z-}$ 3rd instar larvae, probed with antibodies against Ash1 and, as loading control, Caf1. No Ash1 protein is detected in *ash1*$^{22\ z-}$ mutant larvae, Ash1^{R1464A} protein levels are similar to that of Ash1 in *wt* larvae. Asterisk marks an unspecific cross-reacting band that serves as additional loading control.

Development • Supplementary information

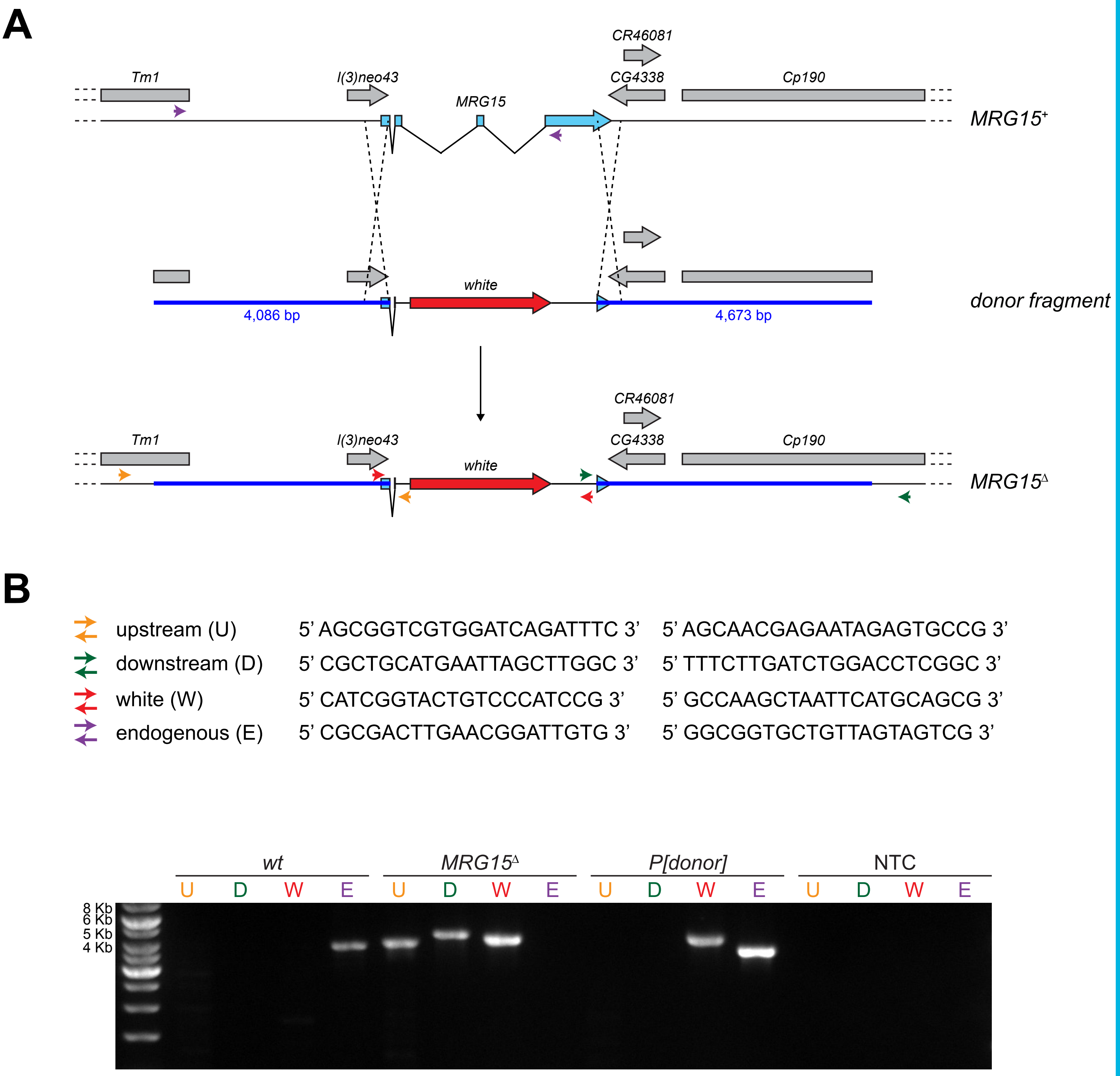

Figure S2

Generation of the *MRG15*$^{\Delta}$ deletion allele.

(A) Diagram of the strategy used to generate *MRG15*$^{\Delta}$.

Top: Schematic representation of the *MRG15* genomic region in *wildtype*. Middle: Donor fragment in the P-element vector with genomic fragments (blue) comprising the homology arms flanking the miniwhite cassette used to replace the *MRG15* gene. Bottom: *MRG15*$^{\Delta}$, the deletion allele obtainedafter ends-out gene replacement by homologous recombination.

(B) Polymerase Chain Reaction (PCR)-based characterization of the *MRG15*$^{\Delta}$ allele. Shown are amplification reactions on genomic DNA from *wt, MRG15*$^{\Delta}$ homozygotes, the *P[donor]* strain harboring the construct with the donor fragment, and a non-template control (NTC) with indicated primer pairs U, D, W and E; location of these amplicons is shown in (*A*).

Development • Supplementary information

Table S1

Complete list of iBAQ values of proteins identified by mass spectrometry in the NTAP-Ash1 and Ash1-CTAP purifications shown in Fig. 1B

Click here to Download Table S1

Table S2

Ash1, Mrg15 and Caf1 peptide sequences from mass spectrometry analysis in Fig. 1B

	Ash-CTAP (in solution digest)	Ash1-CTAP (in gel digest)	NTAP-Ash1 (in gel digest)
**Ash1**			
ADIDADNYQCER		+	
AIQSIKDSYEQQK	+		+
AKEETIVQTAVPR	+	+	+
AEVESPIISAIDIKEDTK	+		+
AIEEGEEITYDYNFSIFNPSEGQPCR	+		
EQAEAAPQPPPKSEPEIRPAK	+		+
CICGIYK		+	
CICGIYKDEGIMIQCSK		+	
CIDAQTAQEQPIDISYIISGR		+	
CMVWQHTECTK		+	
DEGIMIQCSK		+	
DICSAMETIK		+	
DIPIKDESGK		+	+
DISSAVAVAK		+	
DKNIPQYQSTIIQDFMEK		+	
DSPIVPIKVTPPPIIPIEASPDEDVIR		+	+
EEIQIDPIWR		+	+
EETIVQTAVPR		+	
EIDVNKKFR			+
EIPIEEFTEEGHR		+	
EKPVQPVTVEEIPPEIPVSQEEIDAEAEAK		+	+
EQAEAAPQPPPK		+	+
ERDSPIVPIK		+	+
EVDREIPIEEFTEEGHR		+	+
EVISSEEEPGK	+	+	+
EVISSEEEPGKIAVK	+		+
FMTADKGWGVR	+		+
FVNHSCEPNCEMQK	+	+	
FYPNEVVR	+	+	
GGSISATNPDNFISK	+	+	+
GISAPADATAVHVVTPVAPNK	+	+	+
GITQAVHDPEIEKMAK	+	+	+
GRPMECNDEDHCYICEIR	+	+	
GTYIIEYVGEVVTEKEFK	+	+	+
GVIGGKSQR			+
HAVAPGVER			+
HIIEQPTSVSGAGSSASNSPIR	+	+	
HYIITPGERPPAEVAFANGK	+	+	+

Development • Supplementary information

IASYVQIVEIIGDSESIQSFKPK		+	+
IDMAYIDKR	+		+
IGSTAATSKVEFR	+		+
IDSIPTEHDPIPASESHNPGPQDYASCSESSEDK		+	
IENVIITMK		+	+
IIDISPSSICSIK		+	
IITEIEIITSTFNSR		+	+
INAEAWAAAAAAAK		+	+
INESVITK		+	+
INRTGFPTVR			+
IPDGIDPNTNFSCK		+	
IQDDRITGSSGK			+
IQATIAAPSPAQQITINGGGPASTISK		+	
IQPISEKEK			+
IQRPQTPAR			+
ISAIRPTIGVVATK			+
ISVVAIER		+	+
IVVDNNSISGGK	+	+	+
KQKTEIDVGAGPTTMHK	+		
KVSVEQQTTAVIDEHEPEFDPDDEPIQSIRETR	+		
KVVPTVPAPGNSGPAINESADSGVISTTSTTQSTTPSPK	+		
IASSSGISK			+
MQNENAVPTGSIPIASSSKPK	+	+	+
MSDIITTVSSKK	+	+	+
MVIFAK		+	+
MVYTECSPSNCPAGEK	+	+	
NAHKNPAETDSITDQSSQSK	+	+	+
NIEAGTQPK			+
NIPQYQSTIIQDFMEK		+	+
NPAETDSITDQSSQSK		+	+
NREQAEAAPQPPPK		+	
NVVPSWNYR		+	+
QAGKDISSAVAVAK		+	+
QFNTFIVR		+	
QGDAVYVIR	+	+	+
QKTEIDVGAGPTTMHK		+	
QPVIEEPPPTPPPQQK		+	+
QSIMPPPAK		+	+
RVESDTEDTTVEGSFRK		+	
RFYPNEVVR		+	+
RIDSIPTEHDPIPASESHNPGPQDYASCSESSEDK		+	
RPSTPSSPSIAAQISAICSPR		+	
RTEMDFEIPYDIWWAYTNSK		+	
RVESDTEDTTVEGSFR		+	
SATQFSVQR	+	+	+
SDTDGIRMR			+

Development • Supplementary information

SGYVSDYGSVR			+
SINIDSK			+
SIQAQVEQGHYKTPQEFDDHMQQIFVEAK		+	+
SIKSATQFSVQR			+
SIKSATQFSVQRSDTDGIR			+
SMSVGAASGTGASTTICSK	+	+	
SIPTTSASK			+
SQAQFNAR			+
SQSNDSSSPDDHKIPIK(			+
SRIENVIITMK	+		+
SSAASMCSSYVSGVSR		+	
SSADDTVEDQDIIQIAGISIGQSSEESNEYISKPSIK		+	+
SSNNVNVQAAPNPPIDCERVPQAGEAR	+	+	
STASTKSQAQFNAR			+
SYAPHDVDPSIIK	+	+	+
TEIDVGAGPTTMHK	+	+	+
TEMDFEIPYDIWWAYTNSK		+	
TGGNIIIK			+
TNVYAESVRPNIAGFDHPTCNCK		+	
TPQEFDDHMQQIFVEAK		+	+
TQMIGQTVNAK		+	+
TYIVAGIFSNHYK	+	+	
VESDTEDTTVEGSFR			+
VIAAKSGYVSDYGSVR			+
VIYPPPR			+
VKKTYIVAGIFSNHYK		+	
VKPIPAVEAKPSGEGISGR		+	+
VPQAGEARETFVAR			+
VSIYEVVPIEIVIGR	+	+	+
VSVEQQTTAVIDEHEPEFDPDDEPIQSIR	+	+	+
VVETIIHK	+	+	+
WSVNGISR		+	+
YVTTGQYFGR		+	+
YYISIMR		+	+
**MRG15**			
AIPQVAIDINFSKGDR	+	+	
ASTPSKDSNTSQSTASSTPTTSAGPGSK	+		+
DSNTSQSTASSTPTTSAGPGSK		+	
FAIGGGEVIK		+	
EDPAAAETTEEEGPVAPK		+	
GEVKPAKVENYSTGTDANTIFVDGER	+		
GWDVGVAGMK		+	
HPDTPISEIYGSFHIIR		+	
HYITDDWYAVVR		+	
IGPNSTIVFEVEIK		+	

Development • Supplementary information

IIEIPAK	+	+	+
IKIPDEIKHYITDDWYAVVR	+	+	
IQSNNKTFDSIIK	+		
IVDQVVGK		+	
IVDQVVGKGEEAK	+		
MSEQRPSITGSDVAEKPIPPTTTPSTPTTEPAPCVESEEAYAAK		+	
NSSIFFSMSNFINVDPEYVR		+	+
NWDEWVPENR		+	+
QEYIVATVTK		+	
SEAGSTGTTTTNSTANSTTSR	+	+	+
SFHISGVAIDKGQEAK	+	+	
SMFWGINMKPER		+	
TKPDATPVEYYIHYAGWSK	+	+	+
TQYADVMQK	+	+	+
VENYSTGTDANTIFVDGER		+	+
VICFHGPIIYEAK	+	+	
VITCPPHMAYGAR		+	
VSVYYIGR	+		
VTVQQISEQYIAHK		+	+
YSQTIIK		+	
**Caf1**			
APAVGIDIGTTYSCVGVFQHGK		+	
ARFEEINADIFR		+	
ATIDEDNIK	+	+	
EIEGVCNPIITK		+	
ETAEAYIGK		+	
FDDAAVQSDMK		+	
FEEINADIFR		+	
FEISGIPPAPR		+	
GEFGGFGSVCGK		+	
HPSKPEPSGECQPDIR		+	
HWPFEVVSADGKPK		+	
IEIEIK		+	
IEVTYKDEK		+	+
IGEEQSTEDAEDGPPEIIFIHGGHTAK	+	+	+
IHSFESHKDEIFQVQWSPHNETIIASSGTDR		+	
IHVWDISK		+	+
IIIGTHTSDEQNHIIIASVQIPSEDAQFDGSHYDNEK		+	
IINEPTAAAIAYGIDK	+	+	
IIQDIFNGK		+	
IMIWDTR		+	
IVTHFVQEFK		+	
KFDDAAVQSDMK		+	
MKETAEAYIGK	+	+	
NGIESYCFNMK		+	

Development • Supplementary information

NIFTGHTAVVEDVAWHIIHESIFGSVADDQK		+	
NQVAMNPTQTIFDAK	+	+	
PSHTVDAHTAEVNCISFNPYSEFIIATGSADK		+	
QKEIEGVCNPIITK		+	
QTQTFTTYSDNQPGVIIQVYEGER		+	
RIHVWDISK		+	
SDNAAESFDDAVEER		+	+
SINPDEAVAYGAAVQAAIIHGDK		+	
STAGDTHIGGEDFDNR		+	
SVIHDIVIVGGSTR		+	
TFFPEEISSMVITK		+	
TPSSDVIVFDYTK		+	
TTPSYVAFTDTER	+	+	
TVAIWDIR		+	+
TVTNAVITVPAYFNDSQR	+	+	
VDRSDNAAESFDDAVEER	+		
VEIIANDQGNR		+	
VINEEYKIWK			+
WIDANQIADKEEYEHR		+	
YMPQNACVIATK		+	

Table S3

Differential expression of genes in T3 and T2 imaginal discs from *ash1*^{22} homozygous mutants compared to *wildtype*

Click here to Download Table S3

Development • Supplementary information

Table S4

Antibodies

**Specificity**	**Source/Reference**
H3K36me2	Cell Signaling (C75H12)
H3K36me2	Abcam (ab9049)
H3K36me3	Cell Signaling (D5A7)
H4	Abcam (ab10158)
Abd-B	Developmental Studies Hybridoma Bank (1A2E9)
Ubx	Developmental Studies Hybridoma Bank (FP3.38)
Caf1	Gambetta et al, 2009
Ash1	this study

Reference : Gambetta, M.C., Oktaba, K., Müller, J. 2009. Essential role of the glycosyltransferase Sxc/Ogt in Polycomb repression. *Science* **325**: 93-96.

Development • Supplementary information

Table S5: Primers used for ChIP qPCR analysis

Gene	Position	Forward primer (5' to 3')	Reverse primer (5' to 3')
Ubx	+76.9 kb	GCTCGTTGGATCCACTAAACT	TGAGCCGTTAATTGATCGTGAG
	+75.5 kb	TTCGTTCAGCTCCTTGATCG	ACAGACATACACCCGCTACC
	+34.4 kb	GTCCTGGCCAAGGCAAATATT	CGAAAGGAGAACGGAGAATGG
	+0.8 kb	AATTGGTTTCCAGGGATCTGC	ATCCAAAGGAGGCAAAGGAAC
	0	TCCAATCCGTTGCCATCGAACGAAT	TTAGGCCGAGTCGAGTGAGTTGAGT
mth	+2.1 kb	AGACTGATGACGGAAAGCCA	GTCGACGATACGGTGAAGAAC
	+1.6 kb	CGAAGAGTCACACGGTCAAAG	CTAGACAACCGCGAAGAACAG
CG6310	+0.5 kb	GAGGGCATTCATATCACTTCCG	ACCAAGTCATTACCCGGAGTAT
	+0.1 kb	TTTTCCGGTTGACTCTGCTTG	CCTGCTCCTCTATGTCTTCGT
wg	+7.3 kb	GTCCGGATCGTGTACAGTGA	GCTGCATTCGGACTAACTGG
tsh	+6.8 kb	TGCCTCGTCTGTTTTAAGTGC	TTGGTGGATGAGTTGGATGGA
Lam	+1.3 kb	AGTGCGTGGAAACTGAATCG	ACCACGCCTTTTGTCTCTTC
dpr12	+6.2 kb	TCAAGCCGAACCCTCTAAAAT	AACGCCAACAAACAGAAAATG
	+39.1 kb	CCGAACATGAGAGATGGAAAA	AAAGTGCCGACAATGCAGTTA

Positions of the middle nucleotides in the amplified regions are given relative to the corresponding gene transcription start site.

Development • Supplementary information
